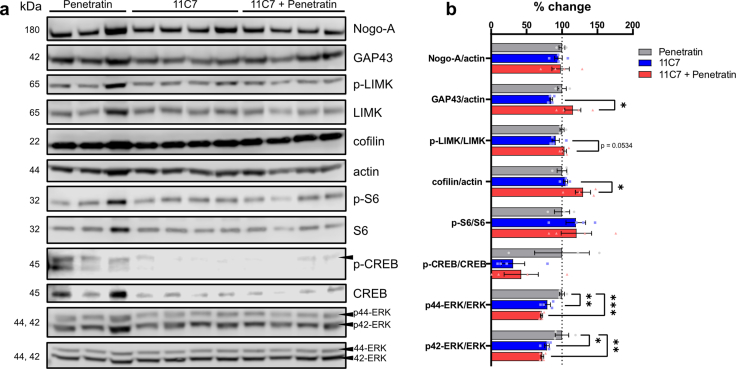# Modulation of antibody transport in the brain and spinal cord through the intranasal pathway

**DOI:** 10.1016/j.neurot.2025.e00606

**Published:** 2025-05-08

**Authors:** Sebastian Spiegel, Sandrine Joly, Ivo Meli, Andrew Chan, Vincent Pernet

**Affiliations:** a Department of Neurology, Inselspital, Bern University Hospital, University of Bern, 3010 Bern, Switzerland; b Experimental Neurology Center (ZEN), Bern University Hospital, University of Bern, 3010 Bern, Switzerland; c Department of Biomedical Research, University Bern, 3010 Bern, Switzerland; d Graduate School for Cellular and Biomedical Sciences, University of Bern, 3010 Bern, Switzerland; e Regenerative Medicine Unit, University Hospital Center of Quebec, Laval University, Quebec City, QC, Canada; f Department of Molecular Medicine, Faculty of Medicine, Laval University, Quebec City, QC, Canada

**Keywords:** Intranasal pathway, Neuronal plasticity, Recombinant antibodies, Blood-brain barrier, Cell-penetrating peptide, Nogo-A

## Abstract

The intranasal pathway is a promising antibody delivery route for the treatment of neurological diseases, but the mechanisms mediating nose-to-brain/spinal cord transport are poorly understood. The aim of our study was to determine if the transport of antibodies can pharmacologically be modulated in the mouse CNS. The pharmacokinetics and distribution of recombinant antibodies were followed in brain and spinal cord homogenates and biofluids by ELISA and immunofluorescence. A non-CNS antigen-binding antibody (FG12) was used to monitor target-independent transport whereas 11C7 mAb, neutralizing the myelin-associated growth inhibitor Nogo-A, was applied to induce CNS target-dependent neuronal growth response. Fast axonal transport/neuronal activity were inhibited with Lidocaine pre-treatment on the olfactory mucosa. Antibody uptake was enhanced across the olfactory epithelium with the co-administration of the cell-penetrating peptide Penetratin. Growth signalling pathways were examined by Western blotting. FG12 was detected in the brain and spinal cord as early as 30 ​min after intranasal administration. After 1 ​h, the concentration of FG12 rapidly declined in all CNS areas and was back to baseline values at 24 ​h. Lidocaine prevented the early rise in FG12 concentration in the spinal cord. This effect was not observed in the brain. Penetratin allowed to maintain the elevation of FG12 and to activate 11C7-induced growth signalling in the spinal cord at 24 ​h. Our data suggest that the pharmacological modulation of transport mechanisms in the nose-to-CNS pathways may allow to control the therapeutic effects of antibodies in neurological diseases.

## Introduction

Antibody-based therapies have emerged as efficient strategies for treating a variety of neurological diseases [[1], [2], [3], [4]]. However, after intravenous injection, only ∼0.01 ​% of antibodies reach the brain [5,6]. This is due to the blood-brain barrier (BBB) and the blood-cerebrospinal fluid barrier (BCSFB) blocking the passage of macromolecules such as antibodies between the blood circulation and the CNS parenchyma [7,8]. To circumvent this issue, relatively high doses of antibodies are needed. To allow better CNS exposure, antibodies can be injected into the cerebrospinal fluid (CSF), within the cerebral ventricles or in the intrathecal space [[9], [10], [11]]. Although effective, these delivery methods are invasive, difficult to put in place in the clinic and associated with an increased risk of infection [12]. In addition, the short half-life of IgG1 antibodies administered in the CSF clearly limits therapeutic effects [[13], [14], [15], [16]].

A promising strategy to bypass the BBB is the application over the nose [[17], [18], [19]]. Preclinical studies using small molecules and peptides, such as Desmopressin [20], Insulin [21,22] and Insulin-like growth factor-I [17], have proven intranasal administration suitable for targeting the CNS. More recently, it has been shown that the use of the intranasal pathway allows to deliver recombinant antibodies with therapeutic effects in the CNS [[23], [24], [25]]. The intranasal administration of a monoclonal antibody (11C7) neutralizing Nogo-A, a potent myelin-associated inhibitor, increased forelimb function recovery in a rat model of stroke [23]. With a refined approach specifically targeting the olfactory mucosa, we reported a fast distribution of 11C7 in the spinal cord of mice in association with motor function improvement after the induction of experimental autoimmune encephalomyelitis (EAE), a model of multiple sclerosis (MS) [25]. The delivery of antibodies to the spinal cord is a major challenge for the treatment of diseases causing lesions predominantly in the spinal cord such as MS [26], neuromyelitis optica spectrum disorder (NMOSD) [27], and myelin oligodendrocyte glycoprotein antibody-associated disease (MOGAD) [28]. However, maintaining antibodies at therapeutic levels is very difficult because of efficient antibody efflux mechanisms [16]. A better knowledge of the mechanisms underlying antibody transport and the identification of factors enhancing the uptake and transport of therapeutic antibodies may greatly contribute to treatment optimization. Here, to follow the transport of antibody in the CNS, we examined the pharmacokinetic of FG12, a recombinant antibody that does not recognize CNS antigens, in contrast to 11C7 whose distribution may strongly be influenced by its binding to endogenous Nogo-A [25]. We tested the effects of Lidocaine, a molecule inhibiting axonal transport/neuronal activity, and Penetratin, a cell-penetrating peptide (CPP) mediating protein transcytosis across epithelial cells, on the pharmacokinetics of intranasally-administered antibodies in the mouse. The two approaches produced opposite pharmacological effects on antibody levels in the spinal cord. On one hand, Lidocaine blocked the early rise of antibody concentration in the spinal cord. On the other hand, Penetratin increased antibody concentration in the brain and in the spinal cord 24 ​h post-administration. Based on these observations, we suggest that axonal transport may control nose-to-spinal cord antibody transfer and that Penetratin co-administration may allow to sustain the delivery of antibodies to the spinal cord.

## Material and Method

### Antibody production

Recombinant HA-tagged FG12 and 11C7 were produced by the Protein production and structure core facility (PTPSP) of the École Polytechnique Fédérale de Lausanne, Switzerland, as described by Joly et al. [29]. Antibodies were concentrated to a final concentration of 10 ​mg/mL using a 30 ​kDa cut-off membrane protein concentrator (Thermo Fisher Scientific, #88521). Hybridoma cells, secreting 11C7, were kindly provided by Prof. Martin Schwab (Univ. Zurich, Switzerland). In brief, cells were cultured in the Turbodoma TP-6 serum free medium (cell culture technologies, Gravesano, Switzerland) supplemented with 0.1 ​% Pluronic F-188 (Sigma Aldrich, P5556, Germany) and 4 ​mM Glutamine under agitation at 37 ​°C in humidified air and 5 ​% CO_2_. Cell culture broth was collected and cleared by centrifugation (30 ​min, 3000×*g*) and sterile filtration using Corning 0.45 ​μm filter cartouches. The purification was performed using a Protein A MabSelect SuRe™ resin (GE Healthcare, Solingen, Germany) on an ÄKTA purifier system (GE Healthcare, Solingen, Germany). Captured IgG were eluted using a 20 ​mM sodium acetate buffer pH 3.0. Subsequently, the elution fractions were introduced to a diafiltration system, tangential flow filtration (TFF) (30 ​kDa cut-off membrane) (GE healthcare, Solingen, Germany). Diafiltration of the antibody was carried out until concentration of 1 ​mg/mL in Phosphate-buffered saline (PBS) pH 6.5. Subsequently, the antibody solution was concentrated to a final concentration of 10 ​mg/mL in PBS pH 6.5 using a vacuum centrifuge. Antibody solution was filtrated at 0.22 ​μm for storage and kept at 4 ​°C. Antibody concentrations were assessed by measuring the absorbance at 280 ​nm, using the Beer-Lambert law (A_280_ ​= ​ε x C x L with A ​= ​absorbance, ε ​= ​molar extinction coefficient in M^−1^cm^−1^ and L ​= ​pathlength in cm) with ε ​= ​276 ​400 ​M^−1^cm^−1^ for 11C7-HA, and ε ​= ​255 ​320 ​M^−1^cm^−1^ for FG12-HA. The purity of antibodies was controlled by SDS-PAGE (data not shown). Mass photometry and dynamic light scattering analyses confirmed the monomeric and monodispersed state of FG12 and 11C7 in PBS (data not shown).

### Animals

Adult female C57BL/6JRj mice of 8 weeks of age were provided by Janvier Labs (France). Mice were housed at the animal facility of the *Zentrum für experimentelle Neurologie/Experimental Neurology Center* (ZEN) at Bern University Hospital (Inselspital). All experiments were carried out in accordance with the guidelines of the Veterinary Office of the Canton of Bern, Switzerland (Licences # BE88/2019, BE32/2023, BE64/2023).

### Intranasal administrations

To limit systemic leakage through the respiratory mucosa, antibodies were selectively applied on the olfactory mucosa of mice, as previously described [30,31]. To do this, a 10-μL Hamilton syringe connected to a 28G neonatal microcatheter (Vygon, 1261.153) was used. To avoid nasal irritation, a balm was applied at the tip of the catheter. In an induction box, mice were anaesthetized for 2 ​min with 3 ​% isoflurane. The catheter was inserted ∼10 ​mm into the nostril. Three μL of antibody solution were administered over 3 ​s. The catheter was kept in place for 5 ​s before withdrawal. The procedure was repeated on the other nostril. After administration, the mouse was placed in a recovery cage. To determine if axonal transport is involved in the transport of antibodies from the nose to the CNS, Lidocaine was used as a reversible inhibitor of fast axonal transport/neuronal activity [[32], [33], [34]]. Mice received 3 ​μL of Lidocaine (Streuli, 10 ​mg/mL) (30 ​μg) in each nostril 10 ​min prior to antibody administration. As a control, 0.9 ​% saline was used.

### Co-administration of Penetratin and antibodies

In order to increase antibody uptake across the olfactory epithelium, L-Penetratin (RQIKIWFQNRRMKWKK, Biosynth®) [[35], [36], [37], [38]] was co-administered at a concentration of 2 ​mM (∼27 μg/animal) with FG12-HA or 11C7 (10 ​mg/mL) in some mice. This dose of L-Penetratin has been shown to be effective to increase the brain uptake of Insulin and Exendin-4 through the nose [35].

### Sample preparations and ELISA

#### CNS homogenate preparation and biofluid collection

Mice were euthanized with an overdose of Pentobarbital 0.5 ​h, 1 ​h, 3 ​h, 6 ​h, or 24 ​h after intranasal administrations. Blood was collected from the right cardiac ventricle. Subsequently, mice were transcardially perfused with PBS to flush blood. CSF was collected through the cisterna magna [6]. CNS tissues were dissected and snap frozen in liquid nitrogen. Plasma was obtained from blood samples after centrifugation at 4000×*g* for 10 ​min at 4 ​°C. CSF was centrifuged at 300×*g* for 5 ​min at 4 ​°C. The supernatant was transferred to a fresh tube and stored at -80 ​°C. CNS tissues were homogenised in lysis buffer (20 ​mM Tris-HCl, pH 8.0; 0.5 ​% CHAPS with anti-protease/anti-phosphatase inhibitors; 100 ​mg wet tissue/400 ​μL lysis buffer) for 1 ​h at 4 ​°C. After 15 ​min of centrifugation at 21 ​130×*g*, 4 ​°C, the lysate supernatant was retrieved in a fresh Eppendorf tube and stored at -20 ​°C.

### ELISA

Antibody concentrations were determined in CNS homogenates or biofluids using ELISA protocols based on HA-tag immunodetection for FG12-HA or the interaction between 11C7 and its Nogo-A_623-640_ epitope. For FG12-HA (Supplementary Fig. 1 A), 50 ​μL of polyclonal rabbit-*anti*-HA-tag antibody (4 ​μg ​mL^−1^, Thermo Fisher Scientific; Cat. No. 71–5500) was added in 50 ​mM carbonate buffer pH 9.6 to high-binding plate wells (Nunc, MaxiSorp, M9410), for 1 ​h at 37 ​°C, and overnight at 4 ​°C. After three washes with PBS-T (0.05 ​% Tween 20), unspecific binding sites were blocked with a blocking buffer containing 5 ​% milk in PBS-T at 37 ​°C for 2 ​h. For the standard curve, FG12-HA was diluted sequentially 1:2 from 100 ​ng ​mL^−1^ to 0.006104 ​ng ​mL^−1^ in a matrix containing either blocking buffer or blocking buffer with CNS lysates from untreated mice. HA-tag detection in CSF was carried out in half area well plates (GreinerBioOne, 7.675 ​061). Coating antibody was incubated at a concentration of 4 ​μg ​mL^−1^ in 25 ​μL of carbonate buffer. Standard curve was prepared in lysis buffer (20 ​mM Tris-HCl, pH 8.0; 0.5 ​% CHAPS with anti-protease/anti-phosphatase inhibitors). CSF samples (2–4 ​μL) were diluted in 25 ​μL of lysis buffer. Tissue lysates of treated mice were diluted 1:2 in blocking buffer (olfactory bulb lysates, dilution 1:5). Samples were incubated for 2 ​h at 37 ​°C. Three washes with PBS-T were followed by a 1 ​h incubation of a cross-absorbed goat anti-mouse IgG-HRP (1:4000 in blocking buffer, Thermo Fisher, Cat. No. 31432) at room temperature. After 3 washes in PBS-T, 3,3′,5,5′-tetramethylbenzidine (TMB) (Thermo Fisher Scientific, N301) was added for 30 ​min at room temperature in darkness to allow colorimetric reaction. The reaction was stopped with equal volumes of 2 ​N/1 ​M sulphuric acid. For 11C7 immunoassay, we used the same ELISA protocol as in our previous studies [25,29]. In brief, biotinylated Nogo-A_623-640_ peptide (SYDSIKLEPENPPPYEEA) (2 ​μg ​mL^−1^ Biosynth®) was added to streptavidin-coated well plate (Thermo Fisher Scientific Pierce, Cat. No. 15125). Unspecific binding was blocked by 5 ​% milk in TBS at pH 7.4 ​at 37 ​°C for 2 ​h. A standard curve was obtained using 25 to 0.0244 ​ng ​mL^−1^ 11C7. Samples were incubated for 2 ​h at 37 ​°C. After three washes in PBS-T, secondary goat-*anti*-mouse IgG-HRP (Millipore, AP127P) was added 1:4000 (0.2 ​ng ​mL^−1^) in blocking buffer for 1 ​h at room temperature. Subsequently, TMB colorimetric reaction was carried out as described for HA-tag ELISA. Absorbance was measured at 450 ​nm using a SpectraMax plate reader (Molecular Devices). The inter-assay coefficient of variation (CV) was calculated using absorbance values at high concentrations (3.125 ​ng ​mL^−1^) and low concentrations (0.006104 ​ng ​mL^−1^). In HA-tag ELISA, the mean CV was ∼4.16 ​%. Analysis, graphical presentation and statistics were performed with the GraphPad Prism 9 software.

### Western blotting

Protein concentration was determine in tissue homogenates with the RC DC protein assay kit (Bio-Rad, Cat. No. 5000121) following the manufacturer's instructions. An equal amount of protein (20 or 60 μg/well) was resolved for each animal by SDS-PAGE in 4–12 ​% Bis-Tris NuPAGE gel (Thermo Fisher Scientific). After migration, proteins were transferred to a nitrocellulose membrane using the Mini Trans-Blot Electrophoretic Transfer cell (Bio-Rad, Cat. No. 1703930). Unspecific binding was blocked by 5 ​% BSA in TBS-T (10 ​mM Tris-buffer saline, pH 7.4, 0.2 ​% Tween20) for 1 ​h at room temperature under agitation. Incubation with primary antibodies was carried out over night at 4 ​°C (see Supplementary Table 1). The day after, the membranes were washed and incubated with the appropriate HRP-conjugated secondary antibody (see Supplementary Table 1).The Licor WesternSure Premium chemiluminescence substrate (LiCor BioScience GmbH, Bad Homburg, Germany) was applied for chemiluminescence detection using the LiCor C-Digit blot scanner (LiCor). The analysis of band intensities was carried out using ImageJ. The GraphPad Prism9 software was used for graphical presentation and statistical analysis of the data.

### Immunofluorescence

Brains and spinal cords were processed to follow the distribution of FG12-HA by immunofluorescence on cryosections after a single intranasal dose of IgG (60 ​μg). PBS-treated mice were used as controls. Deeply anaesthetised mice were perfused with PBS and 4 ​% Paraformaldehyde (PFA). Fixed CNS tissues were cryo-protected in a 30-% sucrose solution. Tissues were embedded in Optimal Cutting Temperature embedding medium (OCT) and frozen on dry ice. Free-floating coronal sections (50 ​μm) were collected in PBS containing 0.01 ​% sodium azide (NaN_3_). For immunofluorescent staining in nasal tissues, bones were decalcified prior to cutting. Skin and muscles were removed thoroughly. Nasal bone decalcification was performed in a PBS solution containing 14 ​% EDTA, pH 8.0. The EDTA solution was changed every other day for 7 days. Decalcified nasal tissues were immersed in OCT. Air bubbles were removed from the nasal cavity under vacuum. Coronal sections (30 ​μm) were directly mounted on glass slides and kept at -80 ​°C until immunostainings. After washes in PBS, slices were incubated in blocking solution (PBS pH 7.4, 5 ​% BSA, 0.3 ​% TritonX-100, 0.01 ​% NaN_3_) for 1 ​h at 37 ​°C, and with HA-tag antibody overnight at 37 ​°C (see Supplementary Table 1). After extensive PBS washes, tissue sections were incubated with goat anti-rabbit IgG AlexaFluor Plus 647 secondary antibody for 4 ​h at 37 ​°C (see Supplementary Table 1). After PBS washes, the sections were mounted with DAPI fluoromount G medium (Bioconcept, 0100–20) on superfrost slides. Images acquired on a Nikon Eclipse Ti-E Epifluorescence microscope, a Zeiss LSM710 and LSM800 (Zeiss, Karlsruhe, Germany). Image analysis was performed with Fiji (RRID:SCR_002285).

### Data and statistical analysis

Statistics were performed with GraphPad Prism 9 (La Jolla, CA, USA). Data are presented as mean ​± ​SEM. Not normally distributed data were compared with Mann-Whitney-test (two-tailed) or Kruskal-Wallis Test with Dunn's multiple comparisons. Normally distributed data were compared using One-Way ANOVA, or unpaired *t*-test. The data and statistical analyses comply with the recommendations on experimental design and analysis in pharmacology.

### Role of funders

This study is part of the Bio-to-Brain Project (Bio2Brain), an EU-funded project of the H2020-MSCA-ITN-2020 (#956977). The funder was not involved in the study design, data collection, analysis, interpretation or reporting.

## Materials

See Supplementary Table 2.

## Results

### Histological examination of the antibody FG12-HA distribution in the nasal cavity and in the brain

The non-CNS antigen-binding antibody FG12 allowed to examine antibody transport mechanisms independently of antigen interactions [39]. FG12 is a mouse IgG1 specific to ethyl *N*-(3,5-dicarboxyphenyl)-*P*-{*N-*[5'-(2″,5″-dioxo-1″pyrrolidinyl)oxy-1′,5′-dioxopentyl]-4aminophenylmethyl}phosphonamidate that has thoroughly been characterized and showed no immunoreactivity in the mouse brain [29]. The uptake of FG12-HA was observed by immunofluorescence in rostral and caudal areas of the nasal cavity after targeted administration onto the olfactory mucosa (Fig. 1a and b). High-magnification images of the olfactory mucosa allowed to detect a bright signal for FG12-HA in typical olfactory sensory neurons and sustentacular cells [40,41], suggesting antibody endocytosis (Fig. 1c–c’, c’’, d). Antibody internalization in OSNs was confirmed with the expression of beta-3-tubulin (Fig. 1 e). In addition, olfactory nerve bundles presented a relatively high level of FG12-HA, suggesting their possible involvement in antibody transport and clearance (Fig. 1 f). In the brain, different structures associated with the olfactory or the trigeminal pathways, such as the piriform cortex (primary olfactory cortex), the hippocampus and the brainstem, showed strong FG12-HA immunoreactivity (Fig. 2 a). Other areas presented intense staining, in particular in the frontal cortex and the inferior colliculus (Fig. 2 a). The localization of FG12-HA in cells of the hippocampus, where the staining was the highest, suggests intracellular antibody transport (Fig. 2 b). Taken together, our histological observations suggest that rapid cell uptake of FG12-HA at the level of the olfactory epithelium and axonal transport may be involved in the CNS distribution of intranasally administered antibodies, independently of antigen recognition.

**Fig. 1:**
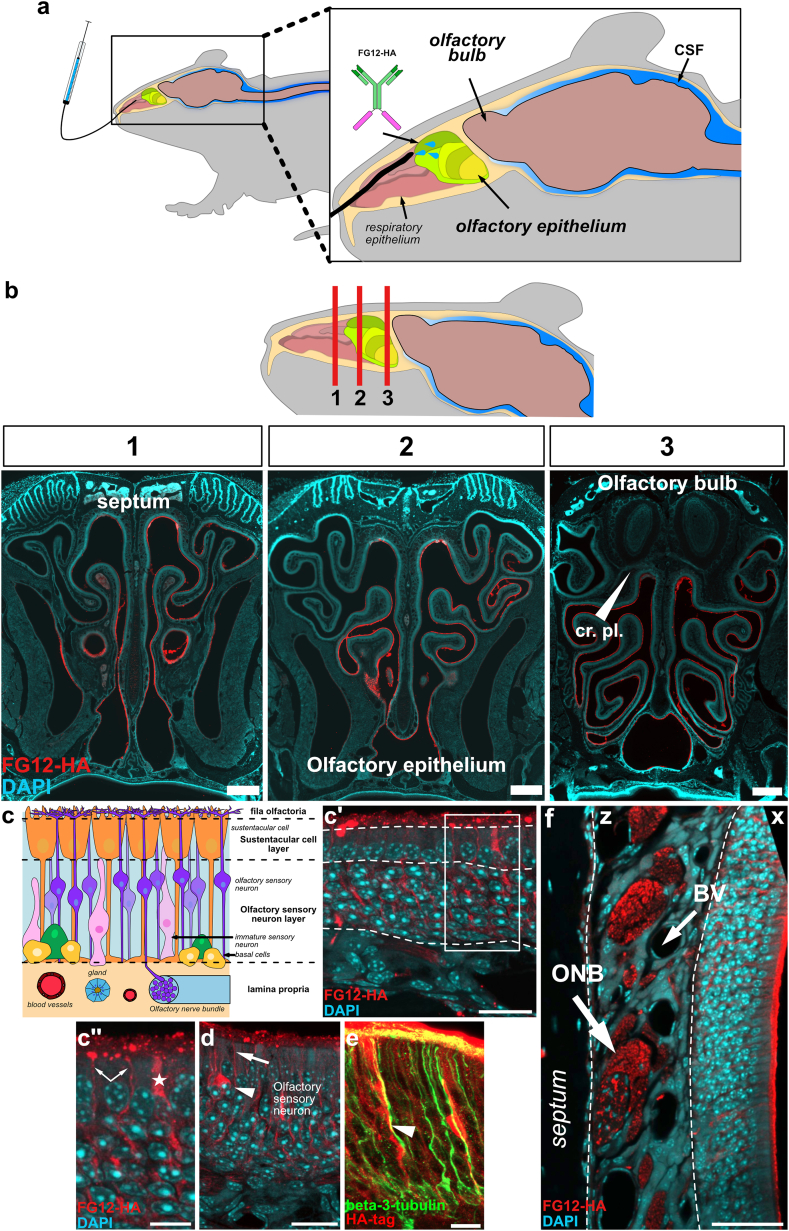
**Histological observation of FG12-HA distribution in the mouse nasal cavity**. **a** Schematic presentation of intranasal administration of non-CNS antigen-binding antibody FG12-HA onto the olfactory mucosa of mice. **b** Distribution of FG12-HA in the nasal cavity 3 ​h post-administration. Cribriform plate (cr. pl.) (n ​= ​3). **c, c’** Confocal images showing FG12-HA uptake at different locations of the olfactory epithelium cells 3 ​h post administration. FG12-HA was observed in the different layers of the olfactory epithelium, that are the fila olfactoria *(f. olfac.*), the sustentacular cell layer, the olfactory sensory neuron layer and the lamina propria. **c’’** Close-up from c' showing FG12-HA in a sustentacular cell (star). Olfactory sensory neuron processes were also positive for FG12-HA (arrow). **d** FG12-HA was also found in the cell body (arrowhead) and neurites (arrow) of olfactory sensory neurons. **e** FG12-HA internalization was observed in beta-3-tubulin(+) olfactory sensory neurons. **f** Detection of FG12-HA in olfactory nerve bundles (ONB) but not in lymphatic vessels and blood vessels (BV) of the lamina propria (z) and the olfactory epithelium (x). Scale bar: 500 ​μm ​(b), 20 ​μm (c’), 10 ​μm (c’‘, d, e) 50 ​μm (f).

**Fig. 2:**
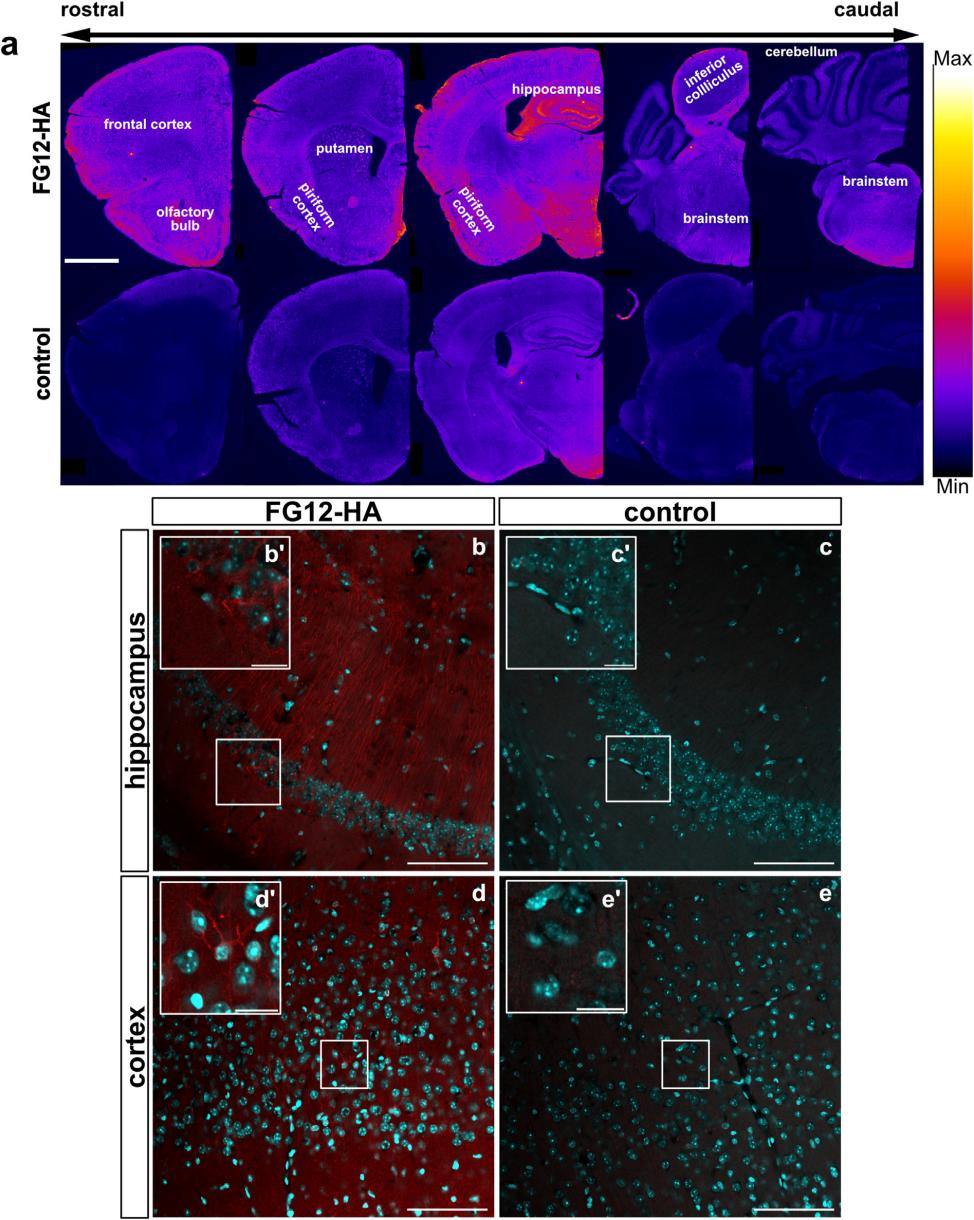
**Distribution of FG12-HA in the CNS after intranasal administration**. **a** Immunofluorescent detection of FG12-HA in the mouse brain (n ​= ​3/group). Colour coded immunofluorescence (Fire colour coding generated with the ImageJ/NIH software) shows staining rostrocaudal variations in brain regions. **b-e** Only mice treated with FG12-HA showed positive cells in the hippocampus (CA1) and in the cortex, compared with control mice left untreated. Close-ups in b and d allowed to distinguish FG12-HA immunoreactivity in individual cells. Close-ups from control mice in c and e exhibited very weak signal. Scale bars: 1 ​mm (a), 50 ​μm ​(b, c, d, e), 10 ​μm ​(b’, c’, d’, e’).

### Pharmacokinetic analysis of FG12-HA in the CNS and biofluids

**Fig. 3:**
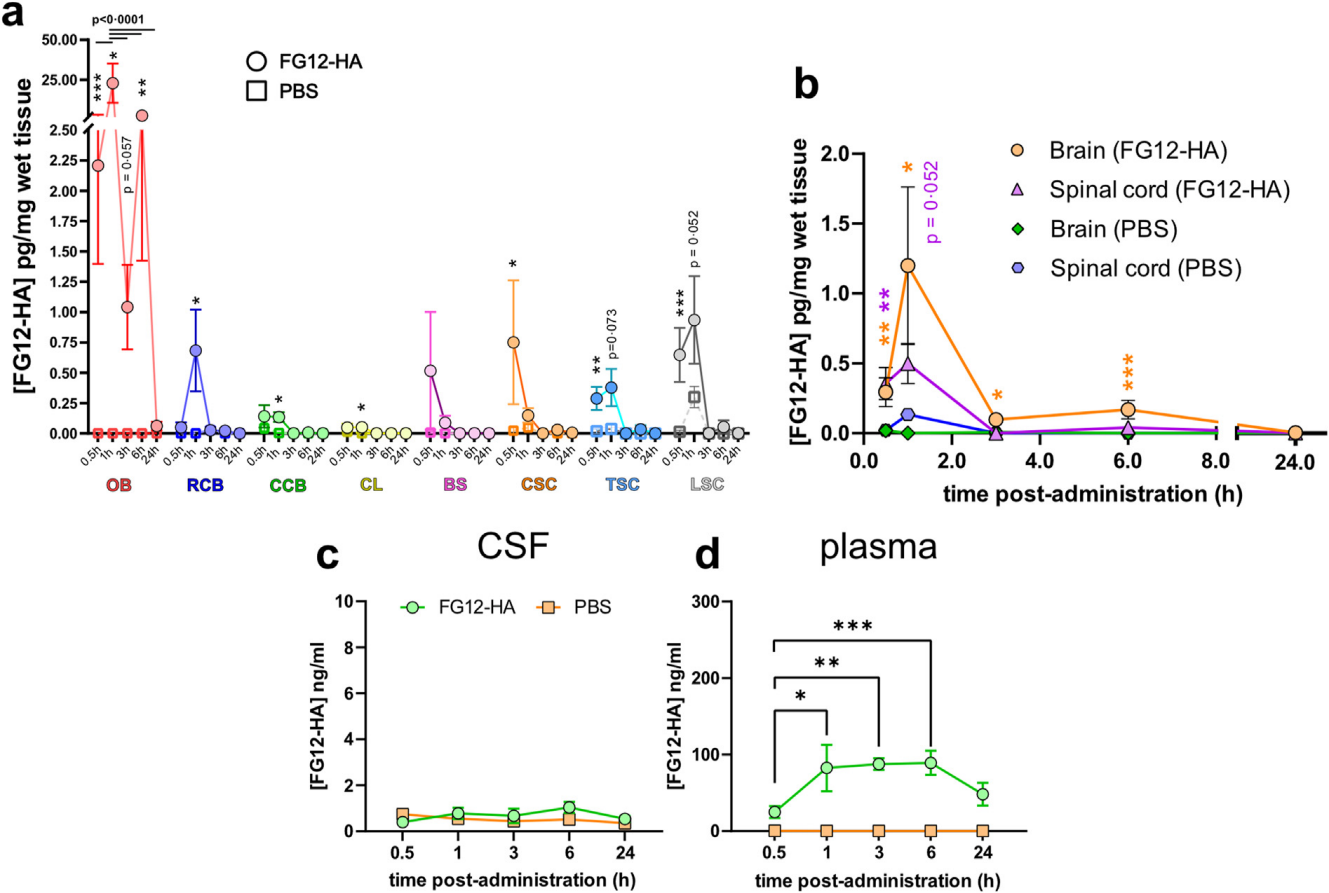
**Pharmacokinetic analysis of FG12-HA**. **a** ELISA detection of FG12-HA in CNS tissues at different time points following intranasal administration. PBS treatment was used as negative control treatment. For region-specific analyses, the CNS was dissected into olfactory bulb (OB, red), rostral brain (RCB, blue), caudal brain (CCB, green), cerebellum (CL, yellow), brainstem (BS, pink), cervical spinal cord (CSC, orange), thoracic spinal cord (TSC, turquoise) and lumbar spinal cord (LSC, grey) pieces. The number of animals per time point was 8 ​at 0.5 ​h, 4 ​at 1 ​h, 4 ​at 3 ​h, 8 ​at 6 ​h and 4 ​at 24 ​h. Statistical test: Multiple Mann-Whitney tests (∗p ​< ​0.05, ∗∗p ​< ​0.01, ∗∗∗p ​< ​0.001). **b** Time-course of FG12-HA variations calculated for the whole brain (OB, RCB, CCB, CL, BS), and spinal cord (CSC, TSC, LSC) tissues, compared to PBS-treated mice. **c** The level of FG12-HA was not significantly different between FG12-HA and PBS treatments in the CSF. **d** In the plasma, the concentration of FG12-HA plateaued from 1 ​h to 6 ​h. Statistical test: Kruskal-Wallis test with Dunn's correction for multiple comparison (∗p ​< ​0.05, ∗∗p ​< ​0.01, ∗∗∗p ​< ​0.001) **a-d**: Data are presented by means ​± ​SEM.

For quantitative analysis, the level of FG12-HA was followed in biofluids, and CNS homogenates by ELISA (Supplementary Fig. 1) after single intranasal administration (60 μg/animal) (Fig. 3). The highest concentration of FG12-HA was detected in the olfactory bulb 1 ​h post administration (Fig. 3 a). In more caudal brain regions, the level of FG12-HA also peaked at 1 ​h. Strikingly, FG12-HA was detected in the 3 spinal cord segments as early as 0.5 ​h post administration (Fig. 3 a). Spinal cord values were lower than in the olfactory bulb but higher than those measured in caudal brain areas. Quantification in the whole brain and spinal cord revealed concentration peaks at 1 ​h, and fast antibody clearance (Fig. 3 b). Of note, a non-significant signal increase was detected at 1 ​h in the spinal cord of mice treated with PBS (Fig. 3 b). The origin of this signal was not identified. In general, the level of FG12-HA was close to baseline values at 3 ​h. FG12-HA was not detectable in the CSF, suggesting limited antibody transfer in, and via the CSF flow (Fig. 3 c). In plasma, FG12-HA concentration was detected 0.5 ​h after intranasal application, and plateaued from 1 ​h to 6 ​h post-administration (Fig. 3 d). FG12-HA kinetics in plasma vs brain/spinal cord suggest direct nose-to-CNS antibody transport, and possible secondary CNS-to-blood release after 1 ​h [5].

### Lidocaine inhibits antibody delivery in the spinal cord

**Fig. 4:**
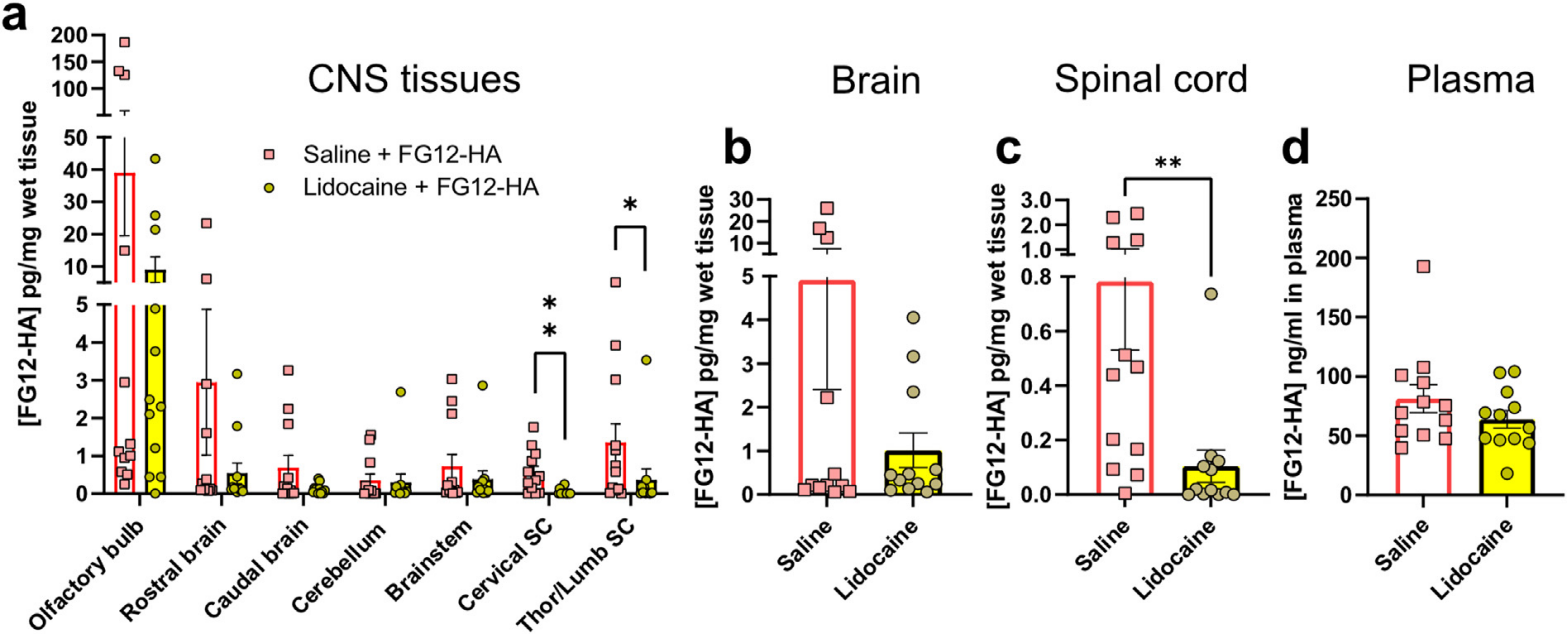
**Lidocaine inhibits FG12-HA transport to the spinal cord**. **a** Changes in FG12-HA concentrations 1 ​h post-administration. Ten min before antibody administration, Lidocaine was applied onto the olfactory mucosa to selectively block fast axonal transport/neuronal activity. In the control group, saline application preceded FG12-HA treatment. Statistical test: Multiple Mann-Whitney test (∗p ​< ​0.05, ∗∗p ​< ​0.01). **b** Lidocaine did not significantly affect FG12-HA concentration in the whole brain. **c** Lidocaine abolished FG12-HA transport to the spinal cord. **d** Plasma FG12-HA concentration was not modified by the intranasal administration of Lidocaine. Twelve mice were used in each treatment group. Bars represent means ​± ​SEM. **b-d** Statistical test: unpaired Mann-Whitney *t*-test (∗p ​< ​0.05, ∗∗p ​< ​0.01).

It has previously been suggested that antibodies may be transported in cranial nerve axons after systemic injections [42]. The detection of FG12-HA in olfactory sensory neurons and their axons in the olfactory tract suggest that neuronal activity and axonal transport in the olfactory nerve may be involved in nose-to-CNS antibody transfer (Fig. 1 d, e). To test this hypothesis, neuronal depolarization/axonal transport were blocked in OSN by applying Lidocaine onto the olfactory mucosa 10 ​min before FG12-HA. Indeed, Lidocaine has been shown to act as a transient inhibitor of fast axonal transport [[32], [33], [34]]. The concentration of FG12-HA was evaluated in CNS areas by ELISA 1 ​h post-administration. The examination of separate brain regions, including the olfactory bulb where FG12-HA concentration used to be the highest (Fig. 3), did not reveal significant changes with Lidocaine vs saline pre-treatment (Fig. 4 a). In contrast, a strong reduction was observed in the cervical, thoracic and lumbar spinal cord regions (Fig. 4 a). At the whole brain level as well, no significant difference was found between the Lidocaine and saline treatment groups (Fig. 4 b). In the whole spinal cord, the very low level of FG12-HA suggests almost complete blockade of antibody delivery (Fig. 4 c). Moreover, the concentration of FG12-HA in plasma remained unchanged in Lidocaine-treated mice compared with the saline control group (Fig. 4 d). These data suggest that neuronal activity/axonal transport contribute to antibody transport between the olfactory mucosa and the spinal cord.

### Enhanced antibody uptake with cell-penetrating peptide

**Fig. 5:**
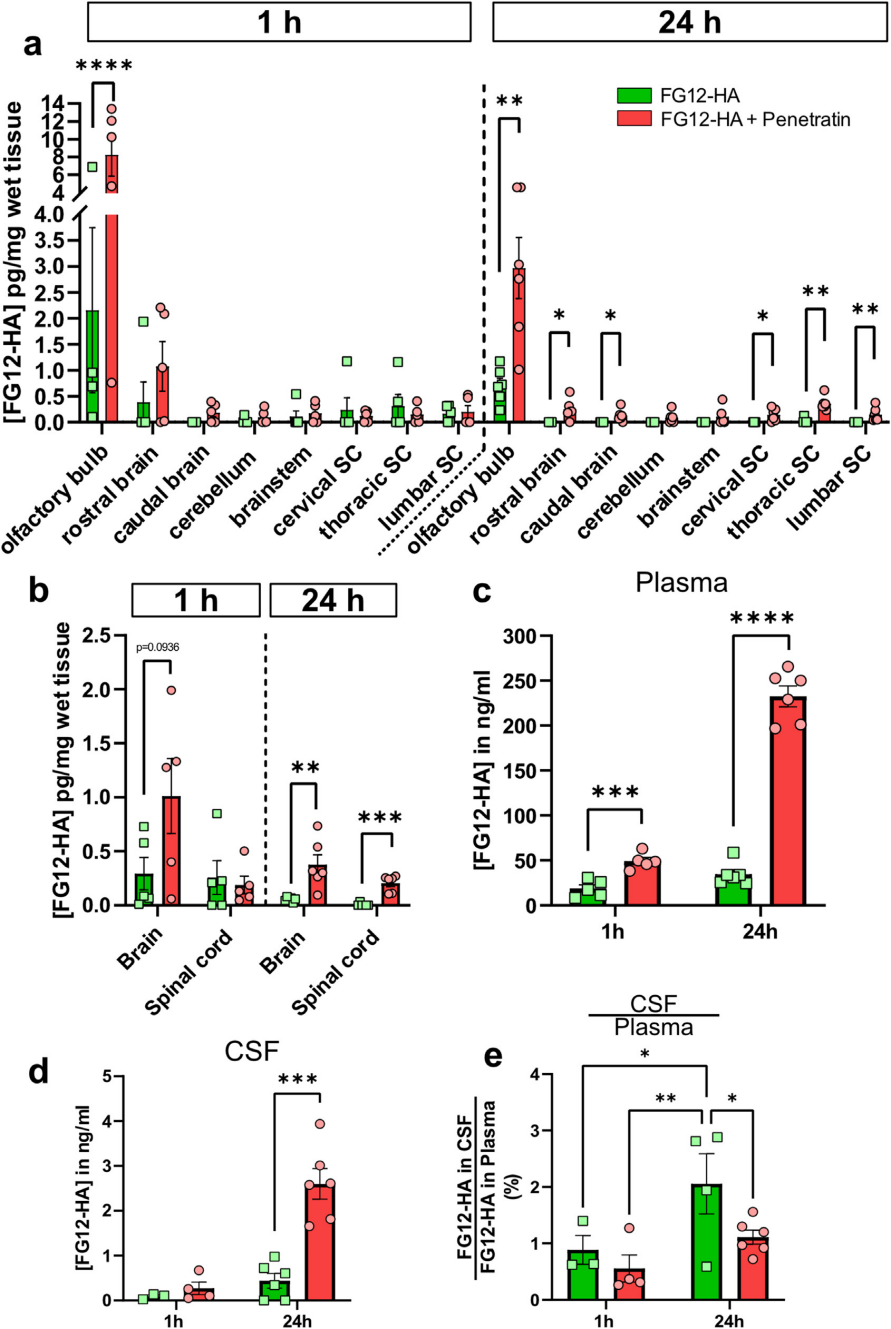
**Enhanced antibody uptake with Penetratin sustains FG12-HA delivery in the brain and the spinal cord**. **a** Co-administration of Penetratin and FG12-HA improved antibody uptake in the olfactory bulb at 1 ​h and in other brain and spinal cord regions at 24 ​h. Five mice were examined at 1 ​h and six mice at 24 ​h (dots represent independent mouse values). **b** At the whole brain and spinal cord levels, Penetratin only significantly increased FG12-HA at 24 ​h. **c** Penetratin increased the concentration of FG12-HA in the plasma at 1 ​h and 24 ​h. ​**d** FG12-HA was only detectable at 24 ​h in the CSF of mice receiving Penetratin. **e** When expressed in % of plasma levels, the concentration of FG12-HA was however not higher with Penetratin. **a-d** Histogram bars represent means ​± ​SEM. Statistical test: Multiple Mann-Whitney tests (∗p ​< ​0.05, ∗∗p ​< ​0.01∗∗∗p ​< ​0.001, ∗∗∗∗p ​< ​0.0001).

To enhance antibody uptake across the olfactory epithelium, Penetratin was co-administered with FG12-HA. Indeed, Penetratin is a small cell-penetrating peptide (CPP), highly soluble in aqueous solution, allowing the transcytosis of co-administered proteins through different epithelia, without side effects and toxicity, in comparison with other CPPs [[43], [44], [45], [46], [47]]. This strategy was successful for small proteins (<6 ​kDa) such as Exendin-4, and Insulin [35,37,[48], [49], [50], [51]]. At 1 ​h, Penetratin only increased the concentration of FG12-HA in the olfactory bulb (Fig. 5 a). In the whole brain, only a trend towards an increase was noticed in the Penetratin group (Fig. 5 b). In contrast, 24 ​h after intranasal application, the concentration of FG12-HA significantly increased in the brain and, to a lesser extent, in the spinal cord of Penetratin-treated mice compared with antibody treatment alone (Fig. 5 b). In the plasma, FG12-HA was more abundant at 1 ​h and 24 ​h, suggesting relatively quick and sustained antibody transfer to the circulation (Fig. 5 c). In the CSF, an increase in FG12-HA was only detected 24 ​h after Penetratin co-administration (Fig. 5 d). However, at this time point, the CSF/Plasma ratio of FG12-HA did not increase in the group receiving FG12-HA ​+ ​Penetratin compared with FG12-HA treatment alone (Fig. 5 e), indicating that the elevation of FG12-HA (Fig. 5 d) may result from plasma-to-CSF transfer. To determine if the increased level of FG12-HA in CNS tissues is attributable to antibody recirculation from plasma, CNS/plasma ratios were calculated (Supplementary Fig. 2). At 24 ​h, the spinal cord/plasma ratio of FG12-HA was higher when Penetratin was co-administered, suggesting direct nose-to-CNS antibody transport with Penetratin, even though plasma-to-CNS transfer cannot be excluded (Supplementary Fig. 2 b). Taken together, our data indicate that Penetratin increases the penetration of antibody in the brain and spinal cord across the olfactory mucosa and further suggest that Penetratin may be used to sustain antibody delivery in the CNS (24 ​h).

**Fig. 6:**
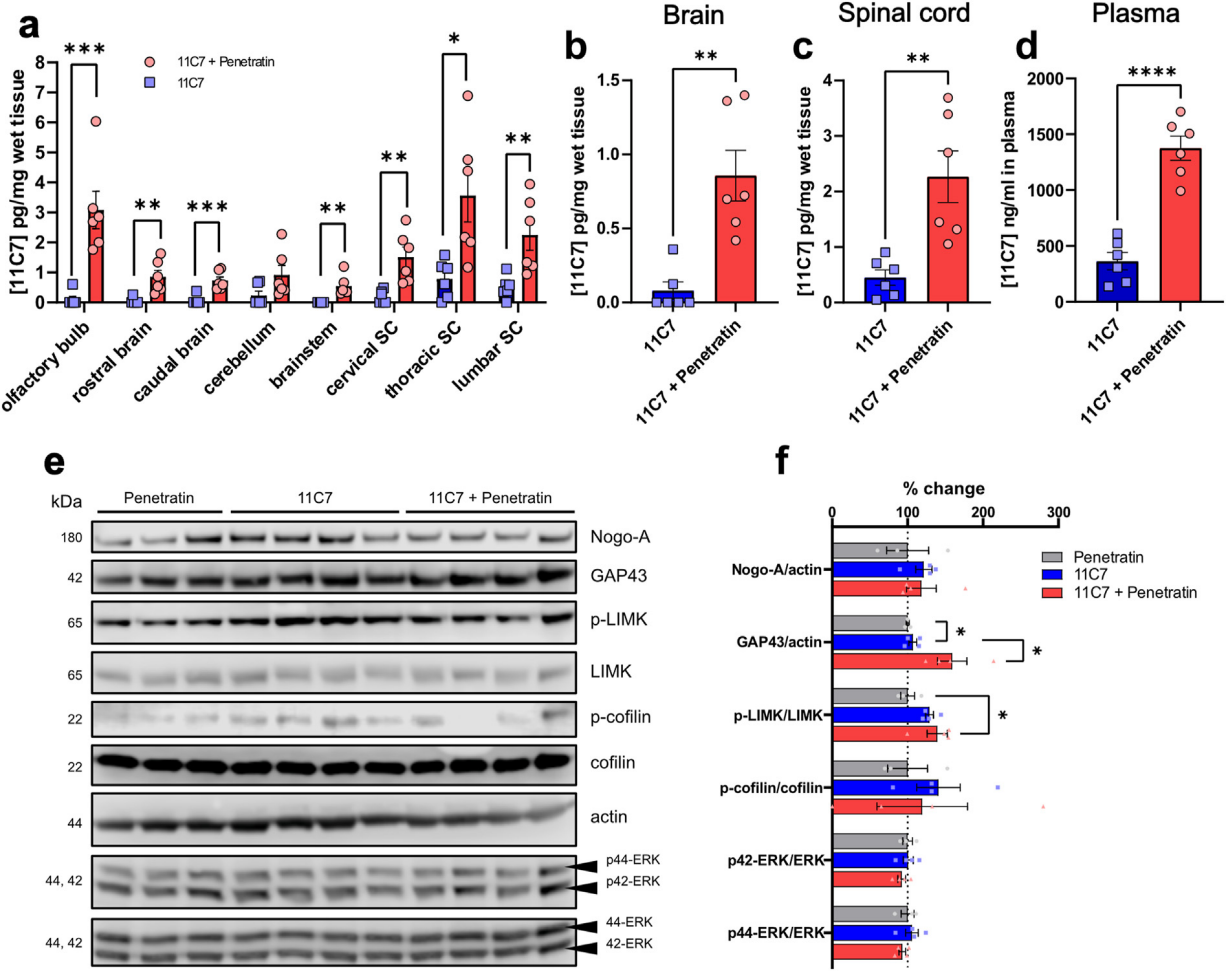
**Penetratin-enhanced 11C7 uptake activates neuronal plasticity mechanisms in the spinal cord**. **a-d** Penetratin strongly increased the level of 11C7 in the brain, spinal cord and plasma. Six mice were used per treatment group. **e** Western blot analysis of Nogo-A signalling in the spinal cord revealed pronounced changes with 11C7 ​+ ​Penetratin compared with 11C7 or Penetratin treatments given separately. Three-four mice were used for each treatment group (1 dot ​= ​1 mouse). Each sample comes from a different animal. **f** Quantitatively, the level of GAP43 was significantly higher in mice administered with 11C7 ​+ ​Penetratin than in those treated only with 11C7 or Penetratin. Only the phosphorylation state of LIMK, involved in Nogo-A signalling, was also statistically different. Protein signals shown in **e** were quantified by densitometry with the ImageJ software and normalized to the average values of Penetratin-treated mice in **f**. Statistical test: unpaired *t*-test (∗p ​< ​0.05). **a-d, f** Data are presented by means ​± ​SEM. **a-d** Statistical test: one way ANOVA (∗p ​< ​0.05).

We then set out to determine if Penetratin-mediated antibody uptake can functionally be relevant in the mouse brain. To address this question, we combined Penetratin with 11C7, a recombinant antibody neutralizing Nogo-A, a potent inhibitor of neuronal plasticity abundantly expressed by oligodendrocytes [25,52,53]. ELISA measurements showed that Penetratin strongly increased the concentration of 11C7 in different brain and spinal cord areas at 24 ​h, a time when the antibody is hardly detectable in mice receiving 11C7 alone, and in plasma (Fig. 6a–d) [25].

To determine if Penetratin-enhanced 11C7 uptake exerted functional effects on growth mechanisms, Nogo-A signalling proteins were followed by Western blot analysis in spinal cord lysates. Among those proteins, growth-associated protein 43 (GAP43) has been shown to increase in the brain and spinal cord after Nogo-A inactivation with 11C7 [54,55]. In addition, changes in LIMK and cofilin phosphorylation in the RhoA/ROCK signalling pathway is a major mechanism mediating the inhibition of neuronal growth by Nogo-A [56]. In KO mice, the inactivation of Nogo-A was associated with the upregulation of phosphorylated LIMK (P-LIMK) and cofilin (P-cofilin) in spinal cord lysates [57]. Moreover, it has been suggested that the phosphorylation of Erk1/2/CREB and P–S6 may act as important effectors downstream of Nogo-A receptor activation in the CNS [29,58,59]. Our data showed that the level of GAP43 was significantly increased in mice receiving Penetratin +11C7 in comparison with those treated with 11C7 only (Fig. 6e and f). In the brain, the levels of GAP43, P-cofilin and P-LIMK were higher with Penetratin +11C7 than with 11C7 (Supplementary Fig. 3a and b). Interestingly, a decreased phosphorylation of P-Erk1/2 was noticed after the co-administration of Penetratin +11C7 when compared with Penetratin but not 11C7 treatment alone. These results suggest that Penetratin may potentiate the blocking effects of 11C7 on Nogo-A signalling and induce the activation of neuronal growth molecules such as GAP43 in the spinal cord.

## Discussion

Our previous data showed that the intranasal pathway is a relevant administration route to treat the spinal cord with therapeutic antibodies in a mouse model of MS [25]. However, the relatively limited knowledge available on the mechanism controlling nose-to-brain, and even more nose-to-spinal cord transport, is a brake to treatment development. In the current study, using FG12-HA, a non-CNS antigen-binding antibody, we observed a similar distribution and pharmacokinetic in brain and spinal cord areas to what we have previously reported for 11C7 [25], in spite of methodological differences (e.g. ELISA methods and use of HA-tagged vs non-tagged antibodies), suggesting that transport mechanisms mediating CNS-wide antibody distribution may be, at least partially, antigen-independent. In addition, the half-life of intranasally-delivered FG12-HA (and 11C7) was very short in the CNS. We showed that the inhibition of neurons with Lidocaine resulted in the virtual abolition of FG12-HA uptake in the spinal cord. In contrast, Penetratin strongly increased the uptake of FG12-HA in the brain and in the spinal cord. This effect was prominent 24 ​h after intranasal administration, suggesting a sustained effect of Penetratin on antibody delivery. Penetratin-induced 11C7 elevation was stronger than for FG12-HA, perhaps as a result of the interaction with its Nogo-A antigen. Changes in Nogo-A signallling activation suggest that CPPs like Penetratin may be used in the optimization of intranasal treatments.

In the nose, the uptake of proteins across the olfactory epithelium may involve paracellular diffusion, intraneuronal transport and transcytosis [17,24,60,61]. The precise mechanisms underlying the transport of antibodies to the brain and spinal cord are elusive. By immunofluorescence, we found that FG12-HA is detected in the olfactory bulb, the hippocampus and the brainstem, which are indirectly connected with the olfactory mucosa via projections from the olfactory bulb and the trigeminal nerve [62,63]. Intracellular staining in the dentate gyrus indicate that FG12-HA is internalized in cells. Since FG12-HA is a non-CNS antigen binding antibody, its intracellular internalization may be mediated by Fc-receptors, that are expressed on microglia cells, and to a lower extent on astrocytes, oligodendrocytes and neurons [[64], [65], [66], [67], [68], [69]]. At this stage, it is not clear whether intracellular FG12-HA localization in brain areas is associated with transport mechanisms. Taken together, our histological observations, showing pronounced FG12-HA staining in OSN cell bodies and their axons, and the dramatic reduction of FG12-HA concentration induced by Lidocaine in the spinal cord, suggest that neurons may be involved in rapid antibody transfer to the spinal cord. Potential axonal transport in the olfactory nerve may not be selective to antibodies, however. Other proteins such as albumin have been detected in olfactory sensory neurons [70]. In addition, axonal transport may occur in different administration routes. For example, it has been shown that axonal transport was required in the sciatic nerve for the uptake of systemically-administered antibodies in DRGs [42,71]. To our knowledge, the effect of Lidocaine on antibody transport after intranasal application has never been reported. Moreover, it would be important to clarify the role of fast axonal transport in the nose-to-brain pathway. Indeed, the effect of Lidocaine in nose-to-brain transport is not entirely clear; the lack of significant changes in the brain of mice treated with Lidocaine may be due to the high variability in antibody uptake that is usually observed after intranasal administration [23,25]. In addition, in this experiment, saline (and Lidocaine) pre-treatment may negatively influence the uptake of FG12-HA and thus produce even more variability between mice. Independently of this important aspect, other mechanisms may contribute to the uptake of antibodies in the olfactory bulb, such as perivascular transport and diffusion. Further investigations are necessary to determine the anatomical pathways through which antibodies travel from the olfactory mucosa and the spinal cord. Indeed, no direct projections exist between the two structures. A mechanism involving axonal transport and transsynaptic transfer of antibody, alone, appears highly unlikely. In mice, the observation of changes in antibody delivery in brain and spinal cord after chemical ablation of the olfactory sensory nerve or trigeminal nerve ligation may allow to clarify the pathway of antibody transport [72]. In addition, other experimental approaches should be tested to validate our results as Lidocaine does not only inhibit axonal transport but can also indirectly influence the blood flow or the glymphatic flow, the contribution of which is not established for antibody transport in the intranasal pathway [73,74]. For example, colchicine is another blocker of axonal transport whose administration may allow to confirm our findings [75]. FG12-HA was not detectable in the CSF with our highly sensitive ELISA method. Consistent with our results, very low levels of antibodies have been found by Kumar et al. in the CSF of rats using radioactive labelling [24]. The CSF may thus play a limited role in the CNS distribution of intranasally-administered antibodies.

Our data showed that Penetratin co-administration increases the uptake of FG12-HA and 11C7 in the whole CNS. At 24 ​h post administration, the CSF/plasma ratio was ∼1 ​%, a level not much different from that obtained after intravenous injection [29], suggesting that the increased level of FG12-HA in the CNS may partially result from plasma-to-CSF transfer. Despite this, a contribution of the nose-to-CNS pathway is expected as the spinal cord/plasma ratio was higher at 24 ​h with Penetratin than after antibody treatment alone. In addition, intravenous injection did not allow us to observe a change in GAP43 expression in the spinal cord ([29], data not shown), in contrast to the intranasal administration of Penetratin and 11C7 in the present study. Therefore, an increased penetration of 11C7 from plasma to CNS is probably not sufficient to explain GAP43 upregulation. The elevation of GAP43 expression may thus be induced through the intranasal pathway. It is also important to underline that our observations were carried out at 24 ​h, a time when the concentration of 11C7 is normally returned to baseline levels. Fast antibody clearance is a major limit to antibody-based treatments in the CNS. Our data support the concept that Penetratin is a suitable cell-penetrating peptides to enhance antibody transport across the olfactory epithelium. Indeed, dose-dependent uptake analyses revealed that transport saturation in the olfactory epithelium restricts the amount of antibody delivered in the brain [23]. To overcome this limitation, matrix metalloprotease-9 (MMP-9) co-administration experiments have been performed [23,24]. However, MMP-9-induced tight junction destabilization may result in olfactory mucosa damage after a single treatment [[76], [77], [78]]. A feature of CPPs like Penetratin is their ability to penetrate cell membranes at low, micromolar concentration without causing significant cell membrane damage [50,51]. Improving the uptake of intranasally applied therapeutic antibodies is essential for the treatment of neurodegenerative diseases such as Alzheimer's and Parkinson's diseases as well as multiple sclerosis. We previously demonstrated that olfactory mucosa-specific administration of 11C7 in the EAE model of MS mitigated motor function impairments [25]. Similarly, Correa et al. [23] showed the improvement of fine motor movement recovery in a rat stroke model after intranasal administration of 11C7. Both studies required daily administrations for 14 or 30 days to maintain therapeutic levels of 11C7 in the CNS. The use of Penetratin may thus allow to keep antibody concentrations in an efficacy range in the CNS, with lower administration frequency. Given the relevance of CPPs demonstrated in clinical trials [79,80], and the relatively weak toxicity of Penetratin in comparison with other CPPs, we think that Penetratin may be a facilitator of antibody uptake in the CNS through the nose [47].

In conclusion, we observed that the uptake of antibodies by olfactory sensory neurons and other cell types in the olfactory mucosa is, at least partially, antigen-independent. Our data suggest that neuronal activity, and perhaps as well fast axonal transport, is required in the transport of intranasally-administered antibodies to the spinal cord. Combined treatments with the cell-penetrating peptide Penetratin may dramatically enhance the therapeutic action of recombinant antibodies in neurological diseases through the non-invasive intranasal pathway.

## Ethics approval and consent to participate

All experiments were carried out in accordance with the guidelines of the Veterinary Office of the Canton of Bern, Switzerland (Licences # BE88/2019, BE32/2023, BE64/2023).

## Availability of data and material

The data supporting the findings of this study are available upon reasonable request to the corresponding author (vincent.pernet@insel.ch).

## Authors contributions

SS, AC and VP designed the experiments and the project, VP and AC supervised the experimental work. SS, SJ, IM, did experiments and analyses. SS, VP and AC interpreted the data. SS and VP wrote the manuscript. SS, SJ, IM, AC and VP reviewed and corrected the final version of the manuscript.

## Declaration of competing interest

AC has received speakers’/board honoraria from Actelion (Janssen/J&J), Almirall, Bayer, Biogen, Celgene (BMS), Genzyme, Merck KGaA (Darmstadt, Germany), Novartis, Roche, and Teva, all for hospital research funds. He received research support from Biogen, Genzyme, and UCB, the European Union, and the Swiss National Foundation. He serves as associate editor of the European Journal of Neurology, on the editorial board for Clinical and Translational Neuroscience and as topic editor for the Journal of International Medical Research.

## Figures and Tables

**figs1:**
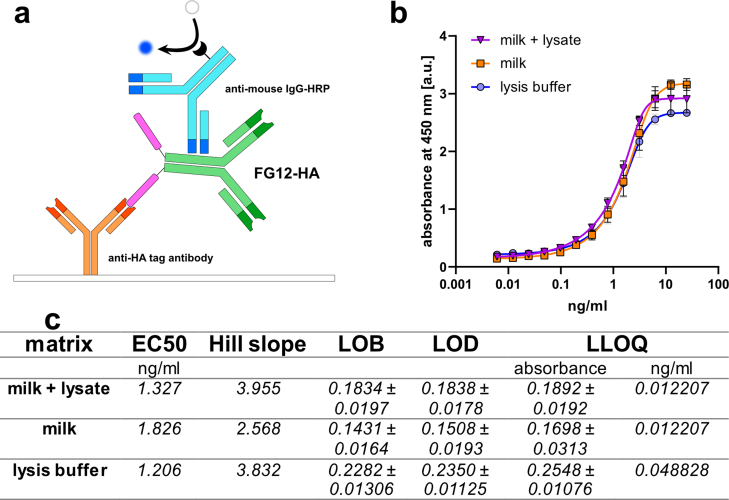


**figs2:**
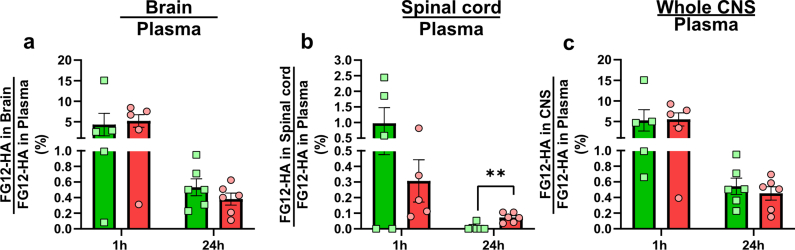


**figs3:**