# Supplementary material for: Modulation of antibody transport in the brain and spinal cord through the intranasal pathway
Source: Neurotherapeutics. 2025 May 8;22(4):e00606. doi: 10.1016/j.neurot.2025.e00606 (PMC12418472; doi:10.1016/j.neurot.2025.e00606)
Supplement: Multimedia component 1 [file mmc1.docx]

***Supplementary Table 1 Antibodies for histology, western blot and ELISA***

| antibodies | host | concentration (mg/mL) | dilution | | | source | |
| --- | --- | --- | --- | --- | --- | --- | --- |
|  |  |  | ELISA | WB | histology | |  |
| Anti-HA tag | rabbit | 0.25 | 1:62.5  (4 µg/mL) | 1:500  (0.5 µg/mL) | 1:200  (1.25 µg/mL) | Thermo Fisher (71-5500) RRID:AB_2533988 | |
| Anti-HA tag | goat | 1 | *n. a.* | *n. a.* | 1:200 | Novus Bio #NB600-362 RRID:AB_10124937 | |
| Goat anti-rabbit IgG Alexa Fluor Plus 647 | goat | 2 | *n. a.* | *n. a.* | 1:200 | Thermo Fisher (A32733) RRID:AB_2633282 | |
| Anti-mouse IgG HRP | goat | 0.8 | 1:4000  (0.2 µg/mL) | n. a. | *n. a.* | Merck Millipore (A127P) | |
| Anti-mouse IgG HRP | goat | 0.8 | 1:4000  (0.2 µg/mL) | 1:10000 | *n. a.* | Thermo fisher scientific (#31432) RRID:AB_228302 | |
| Anti-rabbit IgG-HRP | donkey | 0.8 | *n. a.* | 1:10.000  (0.08 µg/mL) | *n. a.* | Thermo fisher scientific (#31458) RRID:AB_228213 | |
| LIMK | rabbit | *-* | *n. a.* | 1:1000 | *n. a.* | Cell Signaling #3842 RRID:AB_2281332 | |
| P-LIMK | rabbit | 1 | *n. a.* | 1:1000  (1 µg/mL) | *n. a.* | Abcam  (ab38508) RRID:AB_776027 | |
| p-cofilin | rabbit | *-* | *n. a.* | 1:1000 | *n. a.* | Cell Signaling #3313 RRID:AB_2080597 | |
| Cofilin | rabbit | *-* | *n. a.* | 1:1000 | *n. a.* | Cell Signaling #5175 RRID:AB_10622000 | |
| Nogo-A | rabbit | *-* | *n. a.* | 1:10.000 | *n. a.* | Rabbit serum (Bianca) | |
| β-Actin | mouse | *-* | *n. a.* | 1:10.000 | *n. a.* | Sigma Aldrich A5441 RRID:AB_476744 | |
| p-CREB | rabbit | *-* | *n. a.* | 1:1000 | *n. a.* | Cell Signaling # 9198 RRID:AB_2561044 | |
| CREB | rabbit | *-* | *n. a.* | 1:1000 | *n. a.* | Cell Signaling # 9197 RRID:AB_331277 | |
| p-S6 | rabbit | *-* | *n. a.* | 1:1000 | *n. a.* | Cell Signaling # 4858 RRID:AB_916156 | |
| S6 | rabbit | *-* | *n. a.* | 1:1000 | *n. a.* | Cell Signaling # 2217 RRID:AB_331355 | |
| GAP43 | rabbit | *-* | *n. a.* | 1:2000 | *n. a.* | Merck Millipore AB5220 | |
| p-ERK | rabbit | *-* | *n. a.* | 1:1000 | *n. a.* | Cell Signaling # 4370 RRID:AB_2315112 | |
| ERK | rabbit | *-* | *n. a.* | 1:1000 | *n. a.* | Cell Signaling # 4695 RRID:AB_390779 | |
| β-3-tubulin | rabbit |  | *n. a.* | *n. a.* | 1:500 | Abcam #ab18207 RRID:AB_444319 | |

**Supplementary Table 2 List of chemicals, reagents and materials**

| Chemicals | Supplier (article number) |
| --- | --- |
| CHAPS | BioChemica (75621-03-3) |
| EDTA | Sigma-Aldrich (EDS-1kg) |
| Lidocaine | Streuli (QN01BB02) |
| Penetratin | Biosynth (custom) |
| Tris-HCL | Sigma-Aldrich (T3253) |
| Reagents |  |
| anti-protease/anti-phosphatase inhibitors | Thermo Fisher Scientific (78444) |
| Dapi Fluoromount G | Bioconcept (0100-20) |
| Pluronic F-188 | Sigma Aldrich (P5556) |
| RC DC protein assay kit | Bio-Rad (5000121) |
| TMB | Thermo Fisher scientific (N301) |
| TritonX-100 | Sigma-Aldrich (T9284) |
| Turbodoma TP-6 serum free medium | cell culture technologies |
| Tween-20 | Huberlab (A4974.0500) |
| Materials |  |
| 28G neonatal microcatheter | Vygon (1261.153) |
| 30 kDa cut-off membrane protein concentrator | Thermo fisher scientific (88521) |
| Bis-Tris NuPAGE gels | Thermo Fisher scientific (NP0322) |
| Half area high-binding well plate | GreinerBioOne (7.675 061) |
| High-binding well plates | Nunc, MaxiSorp (M9410) |
| Mini Trans-Blot Electrophoretic Transfer cell | Bio-Rad (1703930) |
| Minimate Tangential Flow Filtration Capsules with Omega Membrane, 30k Daltons Pore Size, | Pall (OA030C12) |
| Protein A MabSelect SuRe™ resin | GE Healthcare (17543803) |
| SpectraMax | Molecular Devices |
| Streptavidin-coated well plate | Thermo Fisher Scientific Pierce (15125) |
| Vacuum Filter/Storage Bottle System, 0.45 µm Pore | Corning (430516) |
| Nikon Eclipse Ti-E | Nikon |
| LSM710, LSM800 | Zeiss |
